# Relationships between Mitochondrial Dysfunction and Neurotransmission Failure in Alzheimer’s Disease

**DOI:** 10.14336/AD.2019.1125

**Published:** 2020-10-01

**Authors:** Kan Yin Wong, Jaydeep Roy, Man Lung Fung, Boon Chin Heng, Chengfei Zhang, Lee Wei Lim

**Affiliations:** ^1^School of Biomedical Sciences, Li Ka Shing Faculty of Medicine, The University of Hong Kong, Hong Kong, China.; ^2^Peking University School of Stomatology, Beijing, China.; ^3^Endodontology, Faculty of Dentistry, The University of Hong Kong, Hong Kong, China.

**Keywords:** Alzheimer’s disease, mitochondrial dysfunction, monoaminergic, neurotransmission dysfunction

## Abstract

Besides extracellular deposition of amyloid beta and formation of phosphorylated tau in the brains of patients with Alzheimer’s disease (AD), the pathogenesis of AD is also thought to involve mitochondrial dysfunctions and altered neurotransmission systems. However, none of these components can describe the diverse cognitive, behavioural, and psychiatric symptoms of AD without the pathologies interacting with one another. The purpose of this review is to understand the relationships between mitochondrial and neurotransmission dysfunctions in terms of (1) how mitochondrial alterations affect cholinergic and monoaminergic systems via disruption of energy metabolism, oxidative stress, and apoptosis; and (2) how different neurotransmission systems drive mitochondrial dysfunction via increasing amyloid beta internalisation, oxidative stress, disruption of mitochondrial permeabilisation, and mitochondrial trafficking. All these interactions are separately discussed in terms of neurotransmission systems. The association of mitochondrial dysfunctions with alterations in dopamine, norepinephrine, and histamine is the prospective goal in this research field. By unfolding the complex interactions surrounding mitochondrial dysfunction in AD, we can better develop potential treatments to delay, prevent, or cure this devastating disease.

## 1. Introduction

Alzheimer’s disease (AD) is characterised by progressive cognitive deterioration that manifests with behavioural and psychiatric symptoms [1]. Alzheimer’s disease accounts for 60% to 80% of all dementias [2] and is estimated to affect 115.4 million people worldwide by 2050 [3]. The increasing prevalence of AD and the extreme burdens on individuals and society have led to extensive research into this devastating disease. Despite great efforts over the past few centuries, its molecular origin remains obscure and no treatments can delay or prevent this disease. Since the development of the amyloid cascade hypothesis in the early 1990s, deposition of amyloid plaques in the brain following amyloidosis is considered as one of the leading hypotheses [4]. Some patients with AD have autosomal dominant inherited mutations on amyloid precursor protein (APP), presenilin 1 and 2 genes [5, 6]. In particular, the mutations on presenilin 1 and 2 genes induce subsequent mutations on γ secretase [5, 6]. These eventually lead to defective APP processing, forming Aβ proteins consisting of 42 amino acids [5]. These faulty Aβ proteins are prone to accumulation and account for the plaque deposition leading to neurodegeneration in early-onset familial AD. Interestingly, this familial type only appears in a minority (1%-5%) of the AD population [7].

The majority (more than 95%) of the AD population have the late-onset sporadic type [7]. The well-documented pathological hallmarks of sporadic AD include deposition of Aβ plaques and neurofibrillary tangles of phosphorylated tau (P-tau) [5, 8, 9]. Although the root cause for sporadic AD remains unknown, the most investigated risk factor is apolipoprotein E4 gene, which leads to increased Aβ production and plaque formation [7, 10, 11]. However, the above risk factor and pathological hallmarks are not always found in patients with the sporadic type [7], implying the existence of other causes of AD. Subsequently, a mitochondrial cascade hypothesis was proposed [12, 13], in which mitochondrial dysfunction is a driving force leading to Aβ plaque deposition in AD. In fact, mitochondrial damage was identified as one of the early events in AD pathogenesis [14, 15] and was strongly associated with age-related factors such as non-specific mitochondrial DNA (mtDNA) damage, reactive oxygen species (ROS), and P-tau [16]. Recently, the mitochondrial cascade hypothesis was refined into primary and secondary mitochondrial cascades [17]. The primary cascade is similar to the original hypothesis that mitochondrial dysfunction induces pathological hallmarks such as Aβ and P-tau [13, 17], whereas the secondary cascade posits that mitochondrial dysfunction is an intermediate step majorly initiated by Aβ deposition [17].

Considering the excellent reviews on the associations of Aβ and P-tau with mitochondrial dysfunctions in [17-19], we will only briefly describe their interactions on the role of mitochondrial dysfunction in AD. Both Aβ and P-tau interfere with the electron transport chain, which affects energy production [20, 21], as well as induces mitophagy, leading to excessive loss of mitochondria [22, 23]. Specifically, Aβ causes mitochondrial dysfunctions by arresting transmembrane translocase on mitochondria, interacts with cyclophilin D to hinder the bioenergetics, and facilitates the opening of mitochondrial permeability transitional pores (mPTP) [21, 24]. In addition, Aβ is associated with altered gene expressions that increase mitochondrial fission [21], and amyloid-binding alcohol dehydrogenase contributes to oxidative stress in mitochondria [21, 25]. As for P-tau, it increases dynamin-related protein 1 level, which leads to enhanced GTPase enzymatic activity and excessive mitochondrial fragmentation [26].

Another pathological change in AD patients is the disruption of neurotransmission, which can be observed in the early phases of AD [27, 28] leading to clinical manifestations. Cholinergic and monoaminergic systems have been reported to be altered in AD brains [29]. In particular, the cholinergic system, which regulates memory function and behaviour via the release of the neurotransmitter acetylcholine (ACh) [30], was found to be altered in AD in the 1980s. Accumulation of intraneuronal Aβ in AD degenerates basal forebrain cholinergic neurones and reduces ACh levels [31], which in turn leads to memory deficits [32]. Moreover, monoaminergic systems have also been reported to be defective in AD. Monoaminergic system involve various neurones in the brain that control neurocognitive and neuropsychiatry functions through regulating the release of serotonin (5-HT), dopamine (DA), norepinephrine (NE), and histamine (HA) [33]. Several reports have indicated a significant reduction of 5-HT [34], DA [35] and NE [36] levels as well as their receptors in AD brain, leading to neuropsychiatric and neurocognitive deficits [37]. In AD, loss of 5-HT results in depression, anxiety and agitation [38], whereas dysregulation of DA release leads to reward-mediated memory formation deficits [39], and low level of NE impairs spatial memory function [40]. Unlike the other monoaminergic neurotransmitters, HA showed altered levels in different AD cases [33], which not only leads to changes in learning behaviour, but also neuroinflammation contributing to disease progression [41].

It is very clear that mitochondria and neurotransmission dysfunctions are involved in AD and play an important role in its pathogenesis and clinical symptoms. Understanding the interactions between mitochondrial and neurotransmission dysfunctions are crucial to elucidating the specific mechanisms contributing to the clinical symptoms in AD. Although the associations between mitochondrial dysfunctions and general synaptic deficits were recently reviewed in [18, 42-45], specific interrelationships between mitochondrial dysfunctions and failure in neurotransmission systems is still missing. Therefore, this review aims to examine how mitochondrial dysfunctions affect neurotransmission contributing to AD symptoms, and vice versa, how neurotransmission disruptions, such as in cholinergic and monoaminergic systems, cause mitochondrial dysfunctions.

## 2. Outline of the review

In this review, we first provide a description of mitochondrial dysfunctions in AD, and then discuss the relationships with neurotransmission deficits, how they can trigger one another, and their roles in disease pathogenesis. We identified peer-reviewed articles in the PubMed database and Google Scholar web search engine using the following search terms: ‘Alzheimer*’ AND (‘mitochondria’ OR ‘oxidative stress’) AND (‘neurotransmission dysfunction’ OR ‘acetylcholine’ OR ‘monoamine’ OR ‘serotonin’ OR ‘dopamine’ OR ‘norepinephrine’ OR ‘noradrenaline’ OR ‘histamine’). Additional relevant articles were identified from the reference lists of the included articles, review papers, and book chapters. Only original data were incorporated in this review, whereas review papers were used to provide context and background information.

**Table 1:** Summary of specific types of mitochondrial dysfunctions in Alzheimer’s disease.

Mitochondrial dysfunctions	Evidence	References
Bioenergetic failure		
TCA cycle impairment	Pyruvate dehydrogenase complex ↓	[54, 55, 58]
	Transketolase ↓	[55]
	Alpha-ketoglutarate dehydrogenase complex ↓	[55-58]
	Isocitrate dehydrogenase ↓, Succinate dehydrogenase ↑, and Malate dehydrogenase ↑	[58]
ETC impairment	Complex IV ↓	[20, 59-61]
	Haem-a (structural component of complex IV) ↓	[62-64]
	Transmembrane arrest of TOMM40 & TIM23 pores	[65-67]
	Complex I ↓ due to phosphorylated tau	[20]
	Complex V Dysregulation	[68, 69]
Oxidative stress	Complex IV ↓ with complex III remains intact or ↑	[71]
	Oxidative scavengers (SOD, GPx & GSH) ↓	[72, 73]
	Reactive oxygen species level ↑	[71, 74-76]
	↑Peroxidation of Aβ-bind alcohol dehydrogenase in H_2_O_2_	[25, 77, 78]
	Peroxidation by haem-Aβ complexes ↑	[79, 80]
mtDNA damage		
Specific damage	mtDNA mutations at T477C, T146C & T195C	[107]
Non-specific damage	mtDNA mutations stay at heteroplasmic state and accumulates until energy production impairs	[111, 112]
Ca^2+^ dysregulation	Ca^2+^ influx ↑ from extracellular & endoplasmic reticulum to cytosol upon excitotoxicity	[92, 113-115]
	Ca^2+^ influx ↑ into mitochondria via mPTP	[116]
Defective morphology and dynamics	Fission ↑ with fusion ↓, possibly related to corresponding genes	[108, 119-121]
	Size changes (smaller, spherical, swollen, and/or elongated)	[118, 120, 121]
	Mitochondrial transport to synaptic terminal ↓	[125]
	Cristae ↓ and paler matrix	[120]; Reviewed in [118]
	Mitophagy ↑ due to phosphorylated tau	[22]
Defective mitophagy	PINK1 ↓ and parkin ↓, leading to autophagosomes ↓ and dysfunctional lysosomes ↑	[130]; Reviewed in [22, 23]
Membrane permeabilisation	mPTP opening ↑ with cyt c release	[131, 132]
	Aβ bind to VDAC ↑ leading to mPTP opening ↑	[123]
	TrkA receptor on cell membrane ↓ Extracellular proNGF ↑ Results: pro-apoptotic signalling ↑, and mPTP opening ↑	[135-137]

The ‘↑’ indicates increased level or being stimulated, while ‘↓’ indicates decreased level or being inhibited. Abbreviations: Aβ, amyloid beta; Ca^2+^, calcium ions; cyt c, cytochrome c; GPx, glutathione peroxidase; GSH, reduced glutathione; H_2_O_2_, hydrogen peroxide; mPTP, mitochondrial permeability transition pore; mtDNA, mitochondrial deoxyribonucleic acid; NGF, neuronal growth factor; SOD, superoxide dismutase; TIM23, translocase of the inner membrane 23; TOM40, translocase of outer mitochondrial membrane 40 homolog; TrkA, tropomyosin receptor kinase A; VDAC, voltage-dependent anion channels.

## 3. Mitochondrial dysfunctions in AD

Mitochondria are rod-shaped double membrane intracellular organelles that are the powerhouse of cells. Under aerobic conditions, they produce energy in the form of adenosine triphosphate (ATP) via the tricarboxylic acid (TCA) cycle and electron transport chain (ETC). The ETC oxidises nicotinamide adenine dinucleotide (NADH) and flavin adenine dinucleotide (FADH_2_) from the TCA cycle using oxygen to generate water and ATP, known as oxidative phosphorylation (OXPHOS). In fact, considering 20% of the body’s energy is consumed by the brain [46] and around 93% of all cellular energy is produced by mitochondria [47], the mitochondrion is an indispensable energy source for the neuronal system. Besides providing energy, mitochondria are also involved in intracellular calcium (Ca^2+^) regulation, cellular redox control, and programmed cell death/apoptosis. Many studies have reported that mitochondria appear to be defective in AD. In this section, we review mitochondrial dysfunctions in AD, including impaired cellular energy production, oxidative stress, mtDNA damage, Ca^2+^ dysregulation, impaired mitochondrial dynamics, and mitochondrial membrane permeabilisation (Table 1).

### 3.1 Impaired energy production in mitochondria

As early as the 1980s, reduced glucose metabolism was reported in AD and mitochondrial dysfunction was considered to be a major contributor. Numerous lines of evidence have suggested that glucose metabolism is decreased in the frontal, parietal, temporal, and posterior cingulate cortices of AD patients, possibly due to a disturbed TCA cycle [48-53]. This bioenergetic failure was associated with lower levels of pyruvate dehydrogenase complex (PDHC) [54, 55], transketolase [55], and alpha-ketoglutarate dehydrogenase complex (KDGHC) [55-58], as well as alterations in other TCA cycle enzymes [58], hence, these enzymes are less active in AD leading to hindered energy production by mitochondria.

Furthermore, complexes I and IV (cytochrome oxidase, COX) in the ETC were found to be impaired in AD. The reduced efficacy of COX is the most documented change in AD [20, 59-61], which is associated with (1) a deficiency in its structural complex haem-a [62-64]; (2) transmembrane arrest of translocase of the outer mitochondrial membrane 40 kDa (TOMM40) and translocase of inner membrane subunit 23 (TIM23) protein pore related to Aβ and APP processing [65-67]; and (3) mtDNA damage. Diminished activity of complex I was also reported in AD as a result of P-tau [20]. Besides, dysregulated complex V (also known as ATP synthase) was reported in human with Aβ pathologies and tau transgenic (Tg) mice [68]. Interestingly, Beck et, al [69] emphasised a strong association between dysregulated complex V and loss of oligomycin sensitivity conferring protein (OSCP) and increased formation of mitochondrial permeability transition pores (mPTP) in Tg mice. The mPTP will be discussed in the upcoming section 3.6. Thus, it is reasonable to assume mitochondria are involved in bioenergetic failure in AD.

### 3.2 Oxidative stress induced by mitochondria

Oxidative stress occurs when reactive oxygen species (ROS) accumulates in mitochondria and cytosol. Superoxide anion (O_2_•^-^), hydrogen peroxide (H_2_O_2_), and hydroxyl radical (HO•) normally produced by ETC complex I and III are scavenged by endogenous antioxidants [70], such as superoxide dismutase (SOD), catalase, reduced glutathione (GSH), and glutathione peroxidase (GPx). In patients with AD, COX is inhibited and complex III is either left intact or stimulated resulting in increased levels of ROS [71]. Meanwhile, levels of SOD, GPx, and GSH are decreased in AD, which reduces the anti-oxidative power [72, 73] and aggravates the accumulation of ROS [71], resulting in cellular redox imbalance [74-76].

In addition, mitochondrial-related oxidative stress can be exacerbated by the intramitochondrial Aβ accumulation, Aβ-binding alcohol dehydrogenase [25, 77, 78], and peroxidation of haem-Aβ complexes under H_2_O_2_ [79, 80]. The oxidative stress then triggers lipid peroxidation [81, 82] and protein oxidation [74, 83, 84], which in turn, lead to oxidative dysfunction of key ETC complexes [85], and PDHC and KDGHC of the TCA cycle [86-88]. Oxidative stress also damages nuclear DNA (nDNA) [89-91] and mtDNA, leading to insufficient production of ETC complex subunits [89, 91-93]. The combined effects of lipid peroxidation, protein oxidation and downregulated ETC subunits, lead to bioenergetic failure in AD, which persists or worsens under oxidative stress.

An interesting mechanism relating to oxidative stress was reported to involve the peroxisome proliferator-activated receptor gamma coactivator 1-alpha (PGC-1α) and mitochondrial biogenesis. The PGC-1α, known as a central regulator of metabolism, induces the activities of respiratory complexes, which not only increase ROS production but at the same time increase the content of ROS detoxifying enzymes [94-97]. In cases of AD, the PGC-1α expression is reduced [98-101], implying lower levels of ROS-producing complexes I and III that possibly limit ROS levels in mitochondria. However, this phenomenon also lowers available ROS scavengers, exaggerating the accumulation of ROS. Some studies with ectopic expression have shown rescue of this phenotype in cells and animal models of AD [99, 101, 102], but some studies showed exacerbation of oxidative damage in mice models [103]. Collectively, this raises the question of the precise role of PGC-1α downregulation in inducing oxidative stress in AD. This requires further research to elucidate how and to what extent PGC-1α downregulation mediates biogenesis (ROS production) and ROS detoxification contributing to oxidative damage in AD neuronal mitochondria, as well as which other intrinsic factors of mitochondria synergistically work with PGC-1α to elicit the oxidative damage.

### 3.3 Mitochondrial DNA damage

Mitochondrial DNA, which encodes approximately 99% of polypeptide subunits of ETC complexes [85], has important roles in the vicious cycle of bioenergetic failure involving ETC defects and oxidative stress. Normally, chromatin changes occur in response to DNA damage from oxidative stress and ETC defects [104, 105]. However, mtDNA lacks protective histone and cannot be rescued by post-translational histone modification [93, 106]. As a result, mtDNA is prone to acquiring mutations at mitochondrial control regions such as T477C, T146C, and T195C through mitoepigenetic changes due to its proximity to the ETC [107], leading to impaired assembly and activity of ETC proteins [108, 109]. These ETC impairments lead to mitochondrial energy defects and other pathological changes in AD.

Indeed, mtDNA has a very high mutation rate that is about 10 fold faster than in nDNA, which accounts for the large number of non-specific changes [110, 111]. Mild non-specific damage in mtDNA mostly do not cause severe ETC impairments but coexists with normal mtDNA, which highlights the heteroplasmic state of mtDNA [111]. Deficits in energy production are limited if the proportion of mutant mtDNA remains low [111, 112], but as the proportion of mutant mtDNA increases within the neuron, energy production becomes impaired, leading to mitochondrial dysfunctions and contributing to late-onset sporadic AD [112].

### 3.4 Ca^2+^ Dysregulation in mitochondria

In post-mortem AD brains, mitochondrial Ca^2+^ homeostasis was found to be impaired due to changes in Ca^2+^ signalling [92, 113-115]. This dysfunction can be attributed to excess Ca^2+^ influx from extracellular fluid and Ca^2+^ efflux from the endoplasmic reticulum upon excitotoxicity. Excess Ca^2+^ in the cytosol enters the mitochondria via mPTP altering energy production [116]. In fact, mtDNA mutations and polymorphisms change the intracellular Ca^2+^ handling, leading to Ca^2+^ accumulation in mitochondria altering the mitochondrial matrix pH. Excess cytosolic Ca^2+^ also activates apoptosis in a caspase-dependent manner [92, 117] and contributes to neuronal loss in AD. Furthermore, excess cytosolic Ca^2+^ due to Aβ and P-tau dysregulates KIF5-Miro-Trak-mediated mitochondrial transport to synapses [44]. The defects of mitochondrial dyanmics are discussed in the next section.

### 3.5 Defective neuronal mitochondrial morphology and dynamics

The morphology, dynamics, and motility of mitochondria have also been observed to be altered in AD. Multiple lines of evidence show that neuronal mitochondria in AD have fewer cristae and a paler matrix with enhanced fission and decreased fusion [108, 118-121]. Indeed, oxidative stress and Aβ in AD downregulated genes controlling mitochondrial fusion, such as inner membrane optic atrophy 1, outer membrane mitofusin 1 and mitofusin 2, as well as upregulated fission genes, such as fission 1 and dynamin-related protein 1 [108, 119-121]. Additionally, mitochondrial axonal transport is impaired in AD leading to mitochondrial membrane depolarisation, facilitating retrograde movement of mitochondria [122] damaging synaptic viability [123-125].

### 3.7 Disturbed mitophagy

Healthy cytosolic organelles and proteins are maintained within cells through a clearing process called autophagy, which is initiated by autophagy-related proteins (Atg) [126], and occurs in either selective or non-selective manner [23]. In non-selective autophagy, a double-membraned vesicle, known as autophagosome, engulfs the target along with other content in the cytoplasm to form an autolysosome, which degrades the content by lysosomal proteases [127]. In selective autophagy, nascent autophagosome cargo receptor proteins attach via poly-ubiquitin chains to specific targets for autophagy [128]. Specifically, damaged mitochondria are cleared from the cell by mitochondrial-selective autophagy or mitophagy [129]. Generally, initiation of the mitophagy pathway occurs via the relocation of cardiolipin, a diphosphatidylglycerol lipid, from the inner mitochondrial membrane to outer mitochondrial membrane [23]. Evidence has indicated that two proteins PTEN-induced putative kinase1 (PINK1) and parkin can also initiate the mitophagy pathway leading to autophagosome-mediated mitochondrial degradation [130]. In AD, high levels of Aβ and P-Tau inhibit the expression of PINK1 and parkin, thereby reducing the number of autophagosomes leading to increased dysfunctional lysosomes and the severe disease pathology [22, 23].

### 3.6 Mitochondrial membrane permeabilisation and apoptosis

The unstable mitochondrial membrane acts as a modulator of mitochondrial-mediated apoptosis dependent and independent of caspase, and controls programmed cell death of neurones in AD. The mPTP on the surface of mitochondria are mediated by cyclophilin D [24]. When these pores open, cytochrome c (cyt c) is released from mitochondria into the cytosol, which activates caspases 9 and 3, and induces apoptosis [131-133]. The increased opening of mPTP also disrupts complex V in the ETC, as discussed in section 3.1, in a mechanism involving excess cyclophilin D [69, 134]. The opening of mPTP can be increased by the following factors: (1) oxidative stress; (2) Ca^2+^ accumulation in mitochondria; (3) Aβ binding to voltage-dependent anion channels (VDAC) located on the mitochondrial outer membrane [123]; and (4) extracellular neurotrophic signals, such as neuronal growth factor (NGF) and its precursor, proNGF [135, 136].

In AD brains, there are lower levels of tropomyosin receptor kinase A (TrkA) [135, 136] and higher levels of proNGF [135-137] compared to normal controls. This indicates excess coupling of proNGF to its high-affinity P75 neurotrophin receptor (p75^NTR^) activating the C-Jun N-terminal kinase (JNK)/p53 pathway [138-140]. This pathway further activates B-cell lymphoma-2 (Bcl-2)-associated X protein (Bax), which increases mPTP opening and contributes to the apoptotic pathway.

## 4. Cholinergic and Mitochondrial Dysfunction in AD

Cholinergic transmission is performed by a specific group of neurones at the brainstem and basal forebrain and is involved in cognitive functioning, emotional reactions, memory formation, consolidation, and retrieval [141]. In AD, there is insufficient cholinergic transmission, leading to memory and learning deficits. The decline in cholinergic transmission can result from (1) the loss of cholinergic neurones in the basal forebrain, hippocampus, and amygdala; (2) impairment of ACh metabolism due to imbalanced synthesis and breakdown; and (3) downregulation of ACh receptors (nAChR), except α7 subtype. How these changes are triggered and driven by mitochondrial dysfunctions is discussed in the section below.

### 4.1 Mitochondrial membrane permeabilisation and cholinergic neuronal loss

Discovered in the 1980s, cholinergic neuronal loss in AD was found to be a major contributor to cognitive impairment [142-145]. In AD, there are decreased levels of choline acetyltransferase (ChAT) [146], acetyl-cholinesterase (AChE) [147], and vesicular acetylcholine transporter (VAChT) [148], which are responsible for producing, breaking down, and packing ACh into vesicles, respectively. Neuronal death is tightly correlated with excessive permeabilisation of the mitochondrial membrane, mPTP opening, and release of pro-apoptotic proteins such as cyt c, which activate caspases 9 and 3 to initiate cell death [131-133]. The hyperpermeability of the mitochondrial membrane results in cholinergic neuronal death and leads to ACh insufficiency.

### 4.2 Inhibition of PDHC in mitochondria and ACh synthesis

The ‘bioenergetic failure’ of mitochondria also affects ACh synthesis. Cholinergic neurones synthesise ACh from acetyl coenzyme A (acetyl-CoA) and choline via ChAT in the presynaptic cytoplasm. Most cellular acetyl-CoA is produced in mitochondria from pyruvate in the TCA cycle by the action of PDHC. The majority of mitochondrial acetyl-CoA stays in the mitochondrial compartment and is converted to citrate for energy production [149]. The remaining mitochondrial acetyl-CoA enters the cytosol via temporary release through Ca^2+^-induced mPTP upon depolarisation [150], where ATP citrate lyase (ACL) converts the released citrate into acetyl-CoA for further synthesis [150, 151].

As mentioned above, the activity of PDHC is diminished in AD. Many studies have reported that presence of APP and Aβ peptides cause deficiency in pyruvate utilisation, leading to insufficient mitochondrial and cytosolic acetyl-CoA production for Ach in the cerebral cortex and hippocampus [152-154]. Multiple mechanisms have been proposed to explain this phenomenon. The first mechanism is that accumulating mitochondrial Ca^2+^ activates pyruvate dehydrogenase kinase (PDHK) and inhibits PDHC [155-157]. Meanwhile, excess mitochondrial Ca^2+^ leads to increased mPTP opening, resulting in the release of acetyl-CoA from mitochondria and long-term shortage of ACh and ATP [155, 158]. The second mechanism is that APP and Aβ peptides activate mitochondrial tau protein kinase I/glycogen synthase kinase-3β (TPKI/GSK-3β) pathways [152, 159], leading to dysfunction of lipoic acid, a PDHC cofactor [156]. Therefore, the pathological characteristics of AD hinder the ability of PDHC to convert pyruvate into acetyl-CoA, lowering the efficiency of ACh synthesis.

### 4.3 Mitochondrial-induced oxidative stress and ACh breakdown

Dysfunctional mitochondria can alter the activity of AChE and expression of its gene *ACHE* in the brain, which affects the degree of ACh breakdown. *In vivo* studies have shown that Aβ-induced oxidative stress was associated with enhancements in AChE activity [160, 161]. Moreover, oxidative stress was also found to reduce AChE activity in zebrafish embryo by decreasing the expression of its gene, *ache*[162]. Interestingly, using an AChE inhibitor increased the activity of TCA enzymes and ETC complexes and decreased oxidative stress in AD patients and Tg mice [163, 164]. However, some studies have suggested that this effect might be due to another ligand on AChE inhibitor interacting with Aβ and α7-nAChR rather than through the direct inhibition of AChE [165-167]. Given the vague relationship between mitochondrial oxidative stress and the breakdown of ACh in AD, the causality between the nuclear genome encoding AChE, AChE activity, and mitochondrial dysfunction in AD requires further clarification.

**Figure 1.**
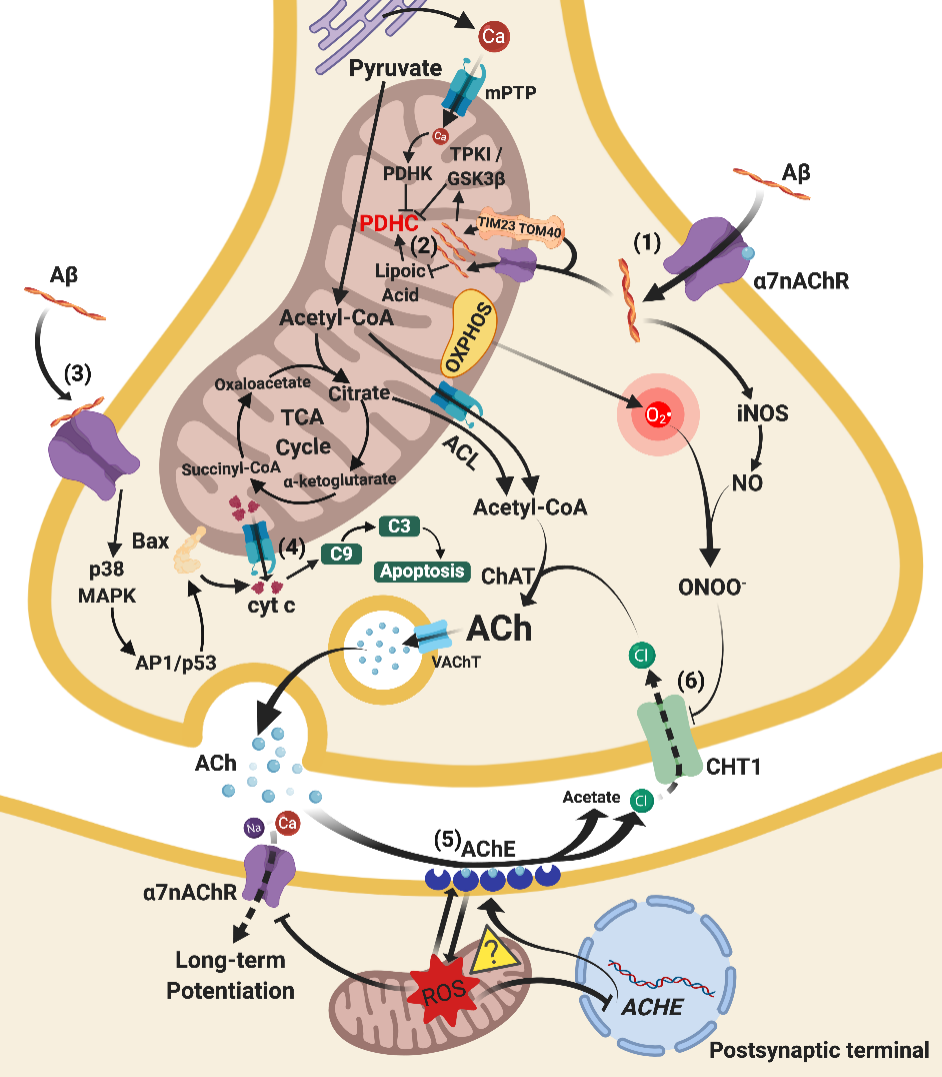
Relationships between mitochondrial and cholinergic dysfunctions. (1) Upregulated α7-nAChRs on cellular and mitochondrial membrane internalise Aβ from the extracellular fluid to the cytosol and intramitochondrial matrix. The cytosolic Aβ induces iNOS production. (2) PDHC is inhibited by Aβ via activation of TPK1/GSK3β and inhibition of lipoic acid and by excess Ca^2+^ influx via activation of PDHK, leading to decreased acetyl-CoA and subsequent ACh synthesis. (3) Upregulated cellular α7-nAChRs combine with Aβ to stimulate p38 MAPK and AP1/p53 signalling pathways. Bax proteins on the mitochondrial membrane are activated and enhance mPTP opening. (4) The mPTP opening on unstable mitochondrial membrane leads to release of cyt c, which activates caspase 9/3-dependent apoptosis. (5) AChE activity is enhanced by oxidative stress from mitochondrial dysfunction possibly through inhibition of *CHAT* nuclear gene. The enhanced AChE activity produces higher ROS levels which damage mitochondria, forming a vicious cycle of lowered ACh levels in the synapse (further investigations are needed). (6) CHT1 is inhibited due to ONOO^-^ produced by O2^-^• from mitochondria and NO from iNOS. Chloride ions reuptake decreases and reduces Ach synthesis. Arrows indicate stimulation, whereas a line with an end bar indicates inhibition. Dotted lines refer to inhibited pathways and question mark in a triangle () represents the need for future studies. Abbreviations: Acetyl-CoA, acetyl coenzyme A; ACh, acetylcholine; AChE, acetylcholinesterase; ACHE, gene that encodes acetylcholinesterase; ACL, ATP citrate lyase; AP-1/p53, activating protein-1 transcription factor / tumour protein p53; Aβ, amyloid-beta; Bax, Bcl-2-associated X protein; C3, caspase 3; C9, caspase 9; Ca, calcium ions; ChAT, choline acetyltransferase; CHT1, choline transporter 1; Cl, chlorine ions; cyt c, cytochrome c; iNOS, induced nitric oxide synthase; p38 MAPK, p38 mitogen-activated protein kinase signalling cascade; mPTP, mitochondrial permeability transition pore; Na, sodium ions; NO, nitric oxide; O2^-^•, superoxide radical; ONOO-, peroxynitrite; OXPHOS, oxidative phosphorylation; PDHC, pyruvate dehydrogenase complex; PDHK, pyruvate dehydrogenase kinase; ROS, reactive oxygen species; TCA cycle, tricarboxylic acid cycle; TIM23, translocase of the inner membrane 23; TOM40, translocase of outer mitochondrial membrane 40 homolog; TPKI/GSK3β, tau protein kinase I / Glycogen synthase kinase-3 beta signalling cascade; VAChT, vesicular acetylcholine transporter; α7nAChR, alpha 7 nicotinic acetylcholine receptor.

### 4.4 Oxidative stress, nitrosative stress, and choline recycling

Mitochondrial-induced oxidative stress via nitrosylative stress hinders the recycling of choline from the synapse leading to ACh deficiency. In late stage AD, levels of presynaptic high-affinity choline transporter 1 (CHT1) were observed to be decreased in synaptosomes in the hippocampus and neocortex of humans [168, 169] and Tg animals [170, 171]. Cuddy et al. demonstrated the internalisation of CHT1 in SH-SY5Y neuroblastoma cells after treatment with nitric oxide (NO) donor (3-morpholinosydnonimine), leading to peroxynitrite (ONOO^-^) formation [171, 172]. SH-SY5Y cells treated with APP were also observed to have internalisation of CHT1 [173, 174], suggesting a connection between APP and ONOO^-^ in choline transport deficits. In fact, APP and its derivatives together with Aβ promoted the formation of inducible nitric oxide synthase (iNOS) leading to NO formation, whereas dysfunctional mitochondria produced excess O_2_•^-^, which combined to form ONOO^-^ causing cellular nitrosative stress. The peroxynitrite-degraded proteasomes, which enhance the internalisation of CHT1, eventually lead to decreased choline reuptake from synaptic clefts [172]. By enhancing nitrosative stress at axonal terminals, the dysfunctional mitochondria restrict the availability of cytosolic choline, another major element for ACh synthesis, resulting in cholinergic dysfunction.

### 4.5 Upregulated α7-nAChR and mitochondrial dysfunction

Normally, muscarinic ACh receptors and nAChR are present on pre- and postsynaptic terminals. Binding of ACh on postsynaptic α7-nAChR stimulates long-term potentiation and facilitates learning and memory formation [175, 176]. High cytosolic oxidative stress inactivates nAChRs and induces rundown of ACh-evoked currents and long-term depression of cholinergic transmission [177], which suggests the oxidative stress produced by postsynaptic mitochondria can alter cholinergic neurotransmission.

The significant upregulation of α7-nAChR at the nucleus basalis of Meynert in AD is possibly due to a compensatory effect from the blocking or disruption by Aβ, which maintains the cholinergic transmission [178-180]. The binding of Aβ to α7-nAchR on the cell membrane stimulates p38MAPK and Bax/Bal pathways to enhance mitochondrial membrane permeabilisation, which increases cyt c release and mitochondrial-dependent apoptosis [178]. On the other hand, agonistic cellular and mitochondrial α7-nAchR can attenuate the p38 MAPK cascade [178], thereby preventing the formation of VDAC and subsequent mPTP opening, as well as mitochondrial-induced apoptosis [181].

Besides its expression on the cell membrane, α7-nAchR also presents on the mitochondrial membrane in parietal cortical and hippocampal cholinergic neurones. The α7-nAchR internalise Aβ from the cytoplasm to the mitochondrial matrix [182, 183] mediated by p38 MAPK, ERK1/2, and low-density lipoprotein receptor-related protein (LRP1) signalling pathways [19, 178, 182-184]. The Aβ accumulation in mitochondria leads to further mitochondrial dysfunctions such as oxidative stress and inhibition of TCA enzymes.

## 5. Monoaminergic Dysfunction and Mitochondrial Dysfunction in AD

Monoaminergic neurotransmission is a diverse network that consists of serotoninergic, dopaminergic, norepinephrinergic, and histaminergic systems. Mitochondrial dysfunctions are associated with monoaminergic inactivity through various mechanisms. In this section, we discuss the common interactions shared between all monoamines and examine specific changes that occur in each system associated with mitochondrial dysfunction in AD.

### 5.1 Monoamine oxidase inhibits mitochondrial functioning

Monoamine oxidases (MAOs), present on the outer mitochondrial membrane of all monoaminergic neurones, are enzymes that catalyze the breakdown of monoamine transmitters. These enzymes are separated into (1) type A (MAO-A), which is extensively expressed in catecholaminergic neurones and eliminate 5-HT, NE, and HA; and (2) type B (MAO-B), which cleave DA [185]. Abnormal MAO activity promotes the loss of monoamines and mitochondrial peroxidation in AD. The activity of hippocampal MAO was significantly increased in humans carrying ε4 allele of apolipoprotein E and in experimental mouse models of AD [186-188]. This enhanced MAO activity not only causes insufficient monoamines for neurotransmission, but also induces significantly higher levels of peroxidative stress in monoaminergic neurones via H_2_O_2_ production. This peroxidative stress causes lipid peroxidation, protein oxidation, nDNA and mtDNA damage, and mitochondrial membrane instability, as well as inhibits the TCA cycle and ETC complexes. Thus, hyperactive MAOs in AD lead to monoaminergic deficiency and mitochondrial dysfunction at presynaptic terminals through peroxidative stress.

In addition, cytoplasmic Ca^2+^ levels affect MAO-A, which alters mitochondrial membrane stability. In a study of glial cell cultures with inhibited PI3K/Akt, ERK and p38 MAPK signalling pathway, free Ca^2+^levels were increased resulting in enhanced MAO-A activity as part of the apoptotic pathway [189, 190]. The high cytosolic Ca^2+^levels in AD lead to more Ca^2+^binding to MAO-A, which increases MAO-A activity and affects mitochondrial membrane stability. Because Ca^2+^homeostasis controls MAO-A, this also modulates mitochondrial permeabilisation and apoptosis. Indeed, artificially enhancing MAO-A by using a dopaminergic neurotoxin (*N*MRSal) increased the mitochondrial membrane potential and induced mPTP opening [191]. Therefore, MAO aggravates mitochondrial dysfunctions through a non-transcriptional pathway.

### 5.2 Serotoninergic and mitochondrial dysfunction in AD

Serotoninergic transmission is mediated through neurones located at the brainstem and basal forebrain [192]. Serotonin or 5-hydroxytryptamine (5-HT) is synthesised from tryptophan catalyzed by tryptophan hydrogenase (TPH) at presynaptic terminals and released into the synaptic cleft. Normally, 5-HT is broken down into 5-hydroxyindoleacetic acid (5-HIAA) under the action of MAO-A. Mitochondrial dysfunction in AD leads to serotoninergic inefficiency via membrane permeabilisation and altered serotoninergic metabolism. Inadequate serotoninergic transmission causes ROS accumulation and further mitochondrial dysfunction, contributing to AD progression.

#### 5.2.1 Mitochondrial membrane permeabilisation and serotoninergic apoptosis

Similar to the cholinergic system, mitochondrial-mediated caspase-dependent apoptosis is one of the causes of serotoninergic neuronal loss. The loss of serotoninergic neurones ultimately decreases 5-HT neurotransmission. Studies in the past 20 years have revealed that patients with AD exhibit extensive loss of 5-HT synthesising neurones in the dorsal and median raphe nuclei [193, 194], and this likely involves mitochondrial membrane permeabilisation and release of pro-apoptotic proteins such as cyt c.

#### 5.2.2 Mitochondrial dysfunction induces excessive 5-HT breakdown

Mitochondrial dysfunctions in AD lead to 5-HT deficits through excessive 5-HT breakdown. In studies using platelets (which can mimic neurones with high APP metabolism and extensive storage of 5-HT) from AD patients and Tg mice, high peroxidative stress (100 mM H_2_O_2_) significantly decreased 5-HT secretion [195]. Indeed, H_2_O_2_ is produced from O_2_•^-^formed in defective ETC in AD. In the cytoplasm of AD neurones, haem-Aβ complex forms when haem is released from mitochondria [196]. Under high peroxidative stress, the haem-Aβ complex breaks down 5-HT to dihydroxytryptamine or tryptamine-4,5-dione [79, 80, 197], which limits the neuroactivity of the serotoninergic system in AD.

#### 5.2.3 5-HT insufficiency induces mitochondrial dysfunction

Inhibiting tryptophan hydroxylase (Tph) and gene knockout in mice to achieve 5-HT deficiency lowered levels of citrate, oxoglutarate, succinate, pyruvate, and antioxidants, which increased oxidative stress [198, 199]. Moreover, dysregulated lipid metabolism was observed in TPH knockout mice, implying that serotoninergic dysfunction might change the lipid composition and induce pathological and functional changes, as well as alter physical properties such as mitochondrial membrane thickness [199]. These results indicate that lower levels of 5-HT were associated with inhibited TCA enzymes and lowered energy production via oxidative stress, but the exact molecular mechanism remains obscure and requires further investigation.

#### 5.2.4 5-HT1A receptors and mitochondrial bioenergetic distribution

The mitochondrial movement in the axons of rat hippocampus are greatly facilitated by the activation of 5-HT1A receptors via stimulation of Akt and inhibition of GSK3β [200]. However, AD neurones have limited binding of 5-HT to 5-HT1A receptors due to downregulated receptors and increased breakdown of 5-HT [201-203], resulting in defective axonal transport of mitochondria. Inadequate binding on 5-HT1A receptors leads to impaired redistribution of energy resources in presynaptic terminals. Therefore, cellular energy production might not meet requirements, compromising global energy distribution and leading to bioenergetic failure in serotoninergic neurones.

#### 5.2.5 Insufficient melatonin and mitochondrial dysfunction

Melatonin possesses anti-oxidative and anti-inflammatory properties, which activates the Bcl-2 pathway to decrease mitochondrial membrane permeabilisation [204, 205], and scavenges ROS to restore cellular oxidative balance [206]. Deficiency of 5-HT in AD decreases the precursor for melatonin production leading to insufficient melatonin. A change in the concentration of melatonin increases mPTP opening, induces oxidative stress, and impairs energy production in neuronal mitochondria. Melatonin treatment was able to rescue the inhibition of ETC and subsequent decrease in ATP production in AD experimental models [207, 208].

**Figure 2.**
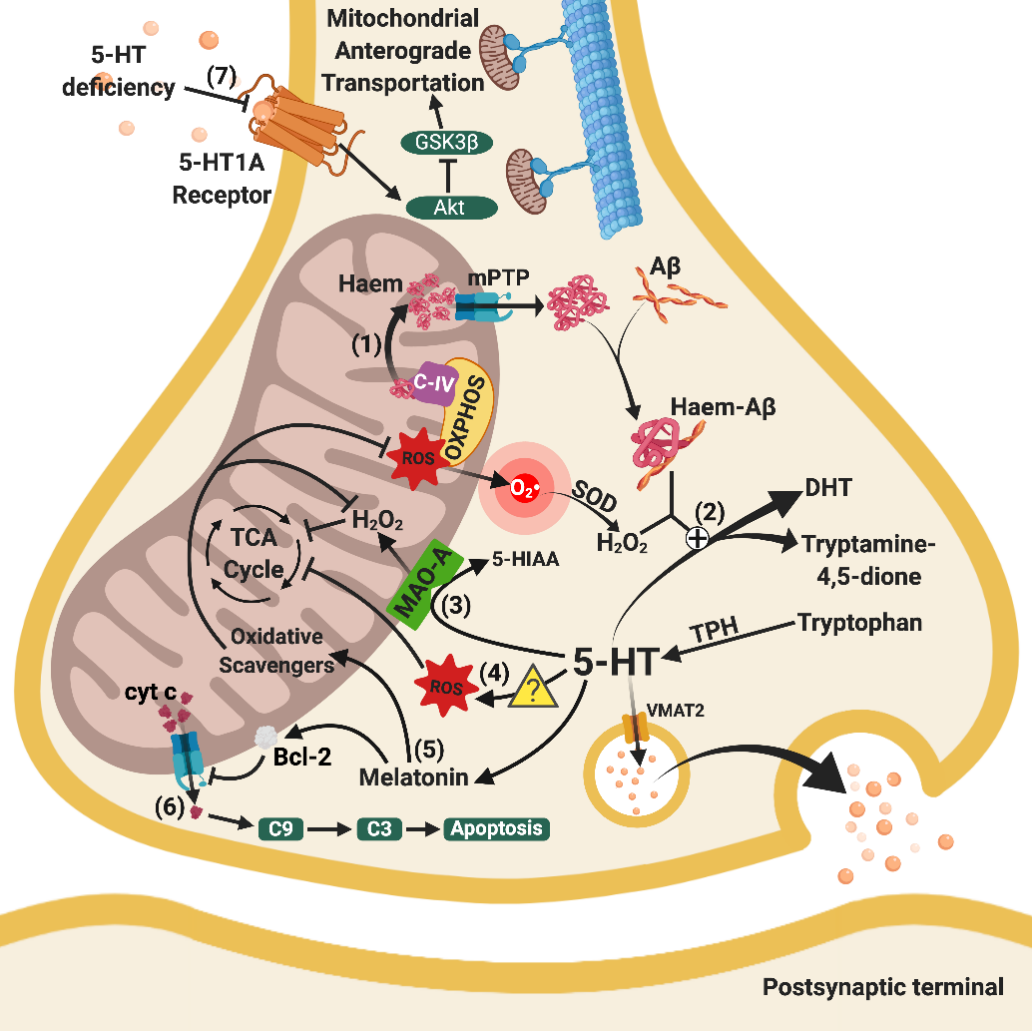
Relationships between mitochondrial and serotoninergic dysfunctions. (1) Haem from C-IV of OXPHOS released from mitochondria through increased mPTP opening bind with cytosol Aβ to form haem-Aβ complexes. (2) Mitochondrial dysfunction enhances the clearance of 5-HT through the combined effects of haem-Aβ complexes and peroxide activity (H_2_O_2_) from defective ETC. (3) Enhanced activity of MAO-A excessively breaks down 5-HT, leading to 5-HT-deficiency and H_2_O_2_, which lowers the efficiency of the TCA cycle. (4) 5-HT is associated with increased ROS levels (further research is needed to elucidate the precise molecular mechanisms), which damage enzymes in the TCA cycle. (5) Loss of 5-HT leads to the loss of anti-oxidative and anti-inflammatory melatonin, indirectly facilitating oxidative damage in mitochondria, reducing the activation of the Bcl-2 pathway. (6) Increased mPTP opening on the mitochondrial membrane leads to the release of cyt c, activating mitochondrial-mediated caspase-activated apoptosis. (7) Decreased 5-HT binding to 5HT-1A receptors hinders mitochondrial anterograde trafficking via inhibition of Akt and subsequent GSK3β stimulation, leading to altered normal energy distribution in the brain. Arrows indicate stimulation, whereas a line with an end bar indicates inhibition. A plus sign in circle (?) refers to catalysation and a question mark in triangle () represents the need for future studies. Abbreviations: 5-HIAA, 5-Hydroxyindoleacetic acid; 5-HT, serotonin; 5-HT1A receptor, serotonin 1A receptor; Akt, protein kinase B; Aβ, amyloid-beta; Bcl-2, B-cell lymphoma 2; C-IV, complex IV in electron transport chain; C3, caspase 3; C9, caspase 9; cyt c, cytochrome c; DHT, 5,7-dihydroxytryptamine; GSK3β, glycogen synthase kinase-3 beta; H_2_O_2_, hydrogen peroxide; MAO-A, monoamine oxidase A; mPTP, mitochondrial permeability transition pore; O2•^-^, superoxide radicals; OXPHOS, oxidative phosphorylation; ROS, reactive oxygen species; SOD, superoxide dismutase; TCA cycle, tricarboxylic acid cycle; TPH, tryptophan hydroxylase; VMAT2, vesicular monoamine transporter 2.

### 5.3 Dopaminergic system and mitochondrial dysfunction in AD

The dopaminergic system consists of a specific group of DA producing neurones, which innervate from the ventral tegmental area (VTA) in the midbrain to ventral striatum and prefrontal cortex, and from the substantia nigra pars compacta (SNpc) to the caudate nucleus and putamen [209]. In AD, there are lower levels of DA in the cingulate gyrus, amygdala, striatum, and raphe nuclei [210], which tightly correlated with the disease severity [211]. This reduction in DA was accompanied by loss of its precursor and metabolites, namely L-3,4-dihydroxyphenylalanine (L-DOPA) and 3,4-dihydroxyphenylacetic acid (DOPAC), respectively [210].

#### 5.3.1 Mitochondrial-dependent apoptosis in the dopaminergic System

The current evidence shows that dopaminergic dysfunction is associated with mitochondrial-mediated apoptosis, specifically excessive mitochondrial membrane permeabilisation. For a long time, it was debated whether the dopaminergic system was involved in the pathogenesis of AD. Several lines of evidence showed that depletion of the VTA volume leads to reduced outflow of DA to the hippocampus and nucleus accumbens. This depletion leads to the deterioration of memory formation and locomotor activities in humans and Tg animals [39, 212-215]. A recent study revealed that selective apoptosis was involved only in the VTA, and not SNpc, hippocampus, neocortex, and locus ceruleus (LC) of Tg mice in the pre-plaque stage of AD [39]. Intriguingly, in Parkinson’s disease, selective apoptosis was found in the SNpc due to mPTP opening and oxidative stress [216]. Given that mitochondrial-mediated selective apoptosis is involved in dopaminergic neuronal loss in the SNpc in Parkinson’s disease, it is highly possible that the apoptosis observed in the VTA in AD shares the same mechanism. Further studies are needed to verify the hypothesis of a pathological connection between mitochondrial-mediated release of pro-apoptotic proteins and dopaminergic neuronal loss in the VTA in AD.

**Figure 3.**
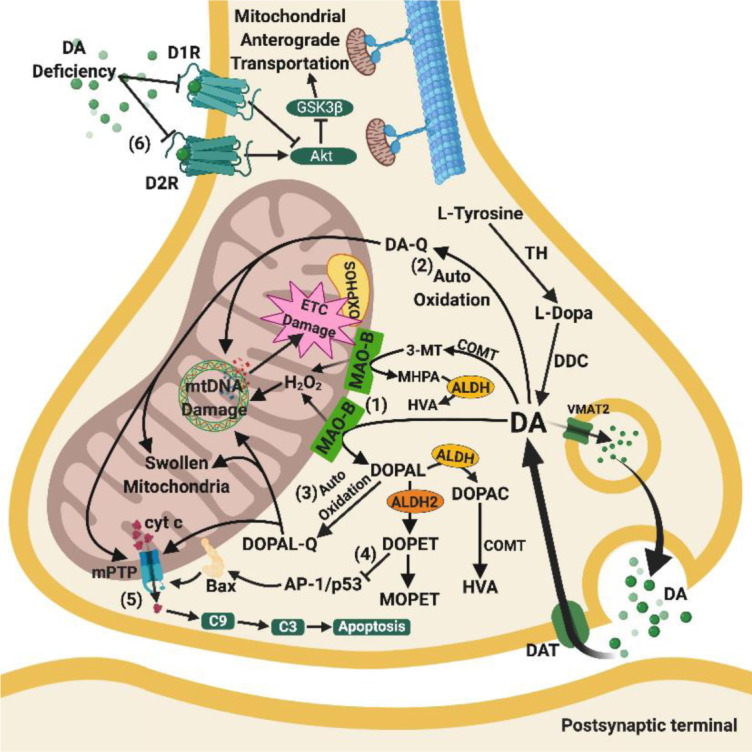
Relationships between mitochondrial and dopaminergic dysfunctions. (1) Overproduction of H_2_O_2_from MAO-B is considered a major peroxidative stressor that damages mtDNA and respiratory complexes of OXPHOS. (2) & (3) DA and DOPAL are auto-oxidised to their respective quinones by ROS formed from OXPHOS. The quinones aggravate the oxidative stress resulting in swollen mitochondria and nuclear DNA damage, and subsequent deficits in OXPHOS complexes. (4) Loss of ALDH2 on mitochondrial surface in AD leads to lower levels of DOPET, which is an anti-apoptotic metabolite cleaved from DOPAL. Loss of DOPET compromises the inhibition of AP-1/P53 and subsequent Bax activation and mPTP opening. (5) DA deficiency also contributes to the inactivation of dopamine 1 and 2 receptors, which control the anterograde transportation of mitochondria. However, investigations are required to elucidate how this loss relates to mitochondrial distribution in the brain. Arrows indicate stimulation, whereas a line with an end bar indicates inhibition. Abbreviations: 3-MT, 3-methoxy-4-hydroxyphenethylamine; Akt, protein kinase B; ALDH, aldehyde dehydrogenases; ALDH2, aldehyde dehydrogenase 2; AP-1/p53, activating protein-1 transcription factor / tumour protein p53; Bax, Bcl-2-associated X protein; C3, caspase3; C9, caspase 9; COMT, catechol-O-methyltransferase; cyt c, cytochrome c; D1R, dopamine 1 receptor; D2R, dopamine 2 receptor; DA, dopamine; DA-Q, dopamine quinone; DAT, dopamine transporter; DDC, DOPA decarboxylase; DOPA, dihydroxyphenylalanine; DOPAC, 3,4-dihydroxyphenylacetic acid; DOPAL, 3,4-dihydroxyphenylacetaldehyde; DOPAL-Q, 3,4-dihydroxyphenylacetaldehyde quinone; DOPET, 3,4-dihydroxyphenylethylamine; ETC, electron transport chain; GSK3β, glycogen synthase kinase-3 beta; H_2_O_2_, hydrogen peroxide; HVA, homovanillic acid; MAO-B, monoamine oxidase B; MHPA, 3-methoxy-4-hydroxyphenylacetaldehyde; MOPET, 4-(2-hydroxyethyl)-2-methoxyphenol; mPTP, mitochondrial permeability transition pore; mtDNA, mitochondrial deoxyribonucleic acid; OXPHOS, oxidative phosphorylation; TH, tyrosine hydroxylase; VMAT2, vesicular monoamine transporter 2.

#### 5.3.2 Contradictory action of DA metabolites and mitochondrial dysfunction

Normally, DA is metabolised into several products including (1) 3-methoxytyramine by catechol-O-methyltransferase (COMT) that then converts to homovanillic acid (HVA) by MAO; (2) 3,4-dihydroxyphenylacetaldehyde (DOPAL) by MAO that then mostly metabolises into DOPAC and HVA by aldehyde hydrogenase and COMT, respectively, and the rest converts to 3,4-dihydroxyphenyl ethanol (DOPET) via alcohol dehydrogenase; and (3) NE by DA beta-hydroxylase (DBH). Besides the loss of DA in AD, there is also loss of its metabolite DOPAC. However, there is a higher concentration of DOPAC per neurone due to hyperactive and upregulated MAO [185]. DOPAC is neurotoxic due to its oxidative properties, which predisposes the mitochondrial structures to damage. In the early 1990s, DA and its metabolites were found to cause DNA damage and inhibit ETC [217]. Later, DA and DOPAC were found to undergo auto-oxidation in the high oxidative environment of mitochondria and MAO to form neurotoxic quinones [218]. Auto-oxidation of DA and DOPAC were found to be correlated with increased quinone content [219] and also its reductases [220] in early AD. These quinones and their derivatives accumulate in the cytosol and add to the oxidative stress [221], leading to architectural changes in the mitochondria including mPTP opening and a swollen morphology [222]. When DA and its derivatives react with heavy metal ions, such as copper ions commonly seen in AD, they non-enzymatically enhance the oxidative damage in DNA and mtDNA by up to 75 fold [223]. These processes aggravate the oxidative stress, damaging neuronal mitochondria in AD.

Surprisingly, DA and its derivatives also elicit neuroprotective effects on mitochondria. They reduce Aβ accumulation and oxidative stress through the upregulation of haem-oxygenase 1 via oxidative transformation under aerobic conditions in microglia [224]. Moreover, another metabolite of DA, DOPET, was found to protect neuronal mitochondria in AD by reversing Aβ-induced mitochondrial-mediated caspase-dependent apoptosis [225]. However, given only a small amount of DOPET is formed under physiological conditions, further evidence is needed. These contradictory effects of DA and its metabolites shed light on how DA and its metabolite levels influence mitochondrial functions in AD.

#### 5.3.3 DA receptors and mitochondrial trafficking

Unlike 5-HT, DA exhibits both stimulating and inhibiting effects on mitochondrial motility via a receptor-dependent mechanism, which affects universal brain energy distribution. Treatment with DA 2 receptor (D2R) agonists decreased mitochondrial transport via enhancing Akt, whereas treatment with DA 1 receptor (D1R) agonists promoted mitochondrial movement via inhibiting Akt [226]. Only the density of D1R was severely reduced in cerebral regions in AD [227], which suggests that inadequate binding on D1R hinders mitochondrial distribution in neurones. However, there are no *in vivo* studies addressing this hypothesis. Further studies are needed to confirm the degree of DA deficiency and subsequent downregulation of receptors in impairing mitochondrial trafficking in dopaminergic neurones in AD.

### 5.4 Norepinephrinergic system and mitochondrial dysfunction in AD

Norepinephrinergic transmission in the brain originates from the locus coeruleus (LC) in the upper dorsolateral pontine tegmentum and projects to the neocortex, hippocampus, thalamus, cerebellum, and spinal cord [209]. In AD, selective neuronal loss at the LC contributes to insufficient NE in hippocampal, temporal, cortical, and cerebellar regions [154, 228], as well as memory and cognitive impairments [40, 229]. The mitochondrial oxidative stress following neuronal activity-dependent Ca^2+^ influx was considered the major contributor to the vulnerability of LC apoptosis in early AD [40].

#### 5.4.1 The contradictory relationship between NE and mitochondrial dysfunction

Under physiological conditions, NE protects neurones from oxidative damage by producing glutathione and peroxisome proliferator-activated receptor delta to enhance the antioxidant system [230, 231]. However, it is worth noting that the neuroprotective effect of NE only lasts a short time [232]. In addition, NE also rescues mitochondrial membrane depolarisation, caspase activation and apoptosis, and maintains the level of mitochondrial aggregation and fusion in Aβ-toxified cells [231, 233]. These additional effects of NE were produced by mediating β-adrenoreceptors, cAMP production, pCREB signalling and subsequent activation of NGF and BDNF pro-survival pathway [233]. The reduction of NE in the LC in AD alters mitochondrial functioning by decreasing oxidative scavengers, such as glutathione, increases oxidative stress, and promotes excessive mitochondrial membrane permeabilisation. The reduced NE was reported to cause excessive oxidative stress and decrease mitochondrial metabolism without activation of nuclear factor erythroid 2-related factor 2, β-adrenoreceptors, and the downstream cAMP/PKA pathway [234].

Norepinephrinergic dysfunction in the cerebellum results in increased ROS production and possibly damage to mitochondrial functioning. In an AD experimental model overexpressing APP, cerebellar NE dysfunction was correlated with upregulation of NAPDH oxidases on the cell membrane [235]. The NADPH oxidases produced more ROS through stimulating Ca^2+^/calmodulin-dependent protein kinases II (CaMKII) and PKCα [235]. The NADPH oxidase-induced CaMKII/PKC pathway activation then led to mtDNA damage and ETC activity through oxidative stress [236]. Although current studies have not reported the precise mechanisms of damage to mitochondria, it is possible that mitochondrial dysfunction might be caused by NE depletion due to loss of norepinephrinergic neurones at the LC.

On the other hand, high concentrations of NE were found to elevate cytosolic and mitochondrial ROS levels [237, 238], which were found to decrease mitochondrial transmembrane potential and energy metabolism in cell culture studies [238], and potentially enhances the neurotoxicity. Given that NE exhibits neuroprotective and neurotoxic effects according to the brain region, neuronal type, and NE concentration, in-depth studies are needed to investigate how NE insufficiency leads to mitochondrial dysfunction in AD according to the location, different stimuli, and NE levels.

**Figure 4.**
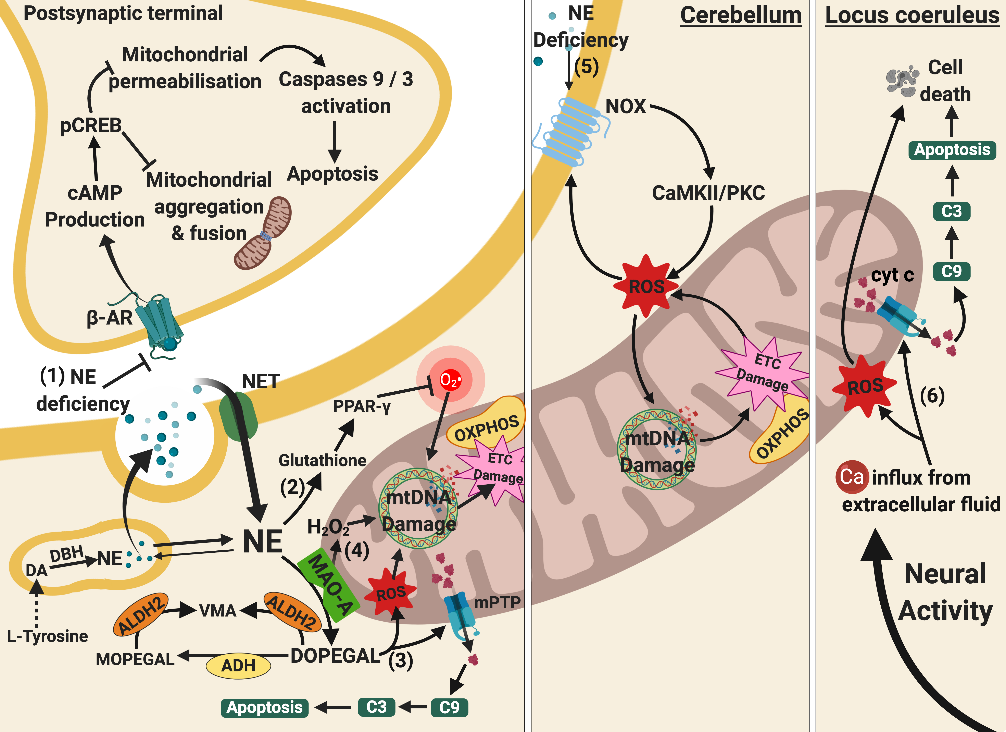
Relationships between mitochondrial and norepinephrine dysfunctions. (1) NE deficiency leads to loss of its protective effects on postsynaptic neurons. Normally, β-AR activation leads to cAMP and pCREB production, reducing mitochondrial aggregation, fission, and membrane permeabilisation. These prevent mitochondrial morphology changes and mitochondrial-mediated caspases-activated apoptosis. (2) NE deficiency at presynaptic terminals lowers the level of glutathione and PPAR-γ activation, increasing O2•^-^ via a receptor-independent pathway and predisposes to mtDNA damage. (3) DOPEGAL accumulates due to undermined ADH and ALDH2, generating both oxidative stress and mPTP opening. The mPTP opening facilitates release of pro-apoptotic cyt c and subsequent apoptosis. (4) Enhanced activity of MAO-A causes overproduction of H_2_O_2_ and along with oxidative stress from DOPEGAL stimulates mtDNA damage and ETC damage. (5) NE deficiency in cerebellum also causes mtDNA damage through the activation of NOX, CAMKII/PKCα signalling cascade, and ROS production. (6) Apoptosis of NE neurones at the locus coeruleus is associated with oxidative stress and mitochondrial-mediated caspases-dependent apoptosis due to Ca^2+^ influx upon neuronal activation, leading to reduced NE production. Arrows indicate stimulation, whereas a line with an end bar indicates inhibition. Dotted line represents a series of biochemical reactions. Abbreviations: ADH, alcohol dehydrogenase; ALDH 2, aldehyde dehydrogenase 2; C3, caspase3; C9, caspase 9; Ca, calcium ions; CAMKII/PKC, calmodulin-dependent protein kinase II/protein kinase C signalling cascade; cAMP, cyclic AMP; cyt c, cytochrome c; DA, dopamine; DBH, dopamine beta-hydroxylase; DOPEGAL, 3,4-dihydroxyphenylglycolaldehyde; ETC, electron transport chain; H_2_O_2_, hydrogen peroxide; MAO-A, monoamine oxidase A; MOPEGAL, 3-methoxy-4-hydroxyphenylglycolaldehyde; mPTP, mitochondrial permeability transitional pores; mtDNA, mitochondrial deoxyribonucleic acid; NE, norepinephrine; NET, norepinephrine transporter; NOX, NADPH oxidase; O2•^-^, superoxide radicals; OXPHOS, oxidative phosphorylation; pCREB, phosphorylated cyclic AMP response element binding; PPAR-γ, peroxisome proliferator-activated receptor gamma; ROS, reactive oxygen species; VMA, vanillylmandelic acid; β-AR, beta-2 adrenergic receptor.

#### 5.4.2 Toxic NE metabolites induce mitochondrial dysfunction

Following DA synthesis, DA is converted into NE in synaptic vesicles via DA beta-hydroxylase (DBH). The majority of NE diffuses out of the vesicles and enters the cytosol for other cellular functions. Cytosolic NE is broken down to 3,4-dihydroxyphenylglycolaldehyde (DOPEGAL) by MAO-A, and further broken down to vanillylmandelic acid (VMA) via aldehyde dehydrogenase 2 (ALDH2) on the outer mitochondrial membrane or by serial reactions of alcohol dehydrogenase (ADH), COMT and ALDH2.

In AD, the activity of MAO-A is enhanced and cleaves more NE into DOPEGAL [239], which is a neurotoxic metabolite that induces various mitochondrial dysfunctions, such as inducing oxidative stress [240, 241] and causing Ca^2+^-induced mPTP opening and apoptosis [239-241]. Ten-fold higher level of DOPEGAL is cytotoxic, causing mitochondrial dysfunction and ATP insufficiency in neurones [242]. In addition, DOPEGAL is formed in close proximity to the outer mitochondrial membrane and can have toxic effects on mitochondria before being broken down by ALDH2 or ADH [239]. In most cases, DOPEGAL accumulates intracellularly due to defective axonal transport at LC axonal terminals [239] and decreased levels of ALDH2 [243, 244]. Furthermore, ALDH2 deficiency in an experimental model of AD disabled the clearance of toxic aldehyde DOPEGAL, resulting in oxidative stress [245, 246] and cascade-dependent apoptosis [246]. Overall, NE dysfunction results in accumulation of DOPEGAL, leading to oxidative damage of mitochondria and inducing excessive mitochondrial membrane permeability.

**Figure 5.**
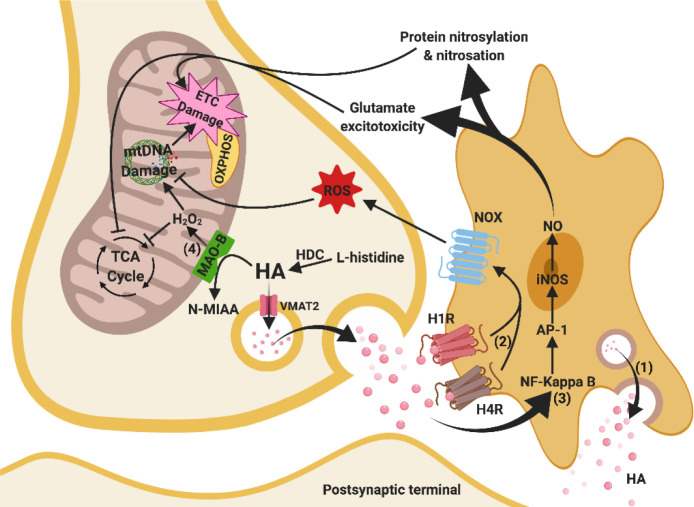
Relationships between mitochondrial and histaminergic dysfunctions. (1) Histamine content in the synaptic cleft increases due to microglial release. (2) Increased HA concentration stimulates H1R and H4R, and subsequent NADPH oxidase, which enhances ROS production and leads to mtDNA damage and ETC damage. (3) Increased HA content at the synapse also induces the NF kappa-B/AP-1 signalling pathway facilitating iNOS synthesis of NO, which in turn leads to glutamate excitotoxicity, nitrosylation and nitrosation of proteins, particularly in OXPHOS (ETC damage) and TCA cycle. (4) Excessive H_2_O_2_produced from upregulated MAO-B leads to damage in mtDNA and the TCA cycle. These mechanisms ultimately damage mitochondrial bioenergy production in the brain. Arrows indicate stimulation, whereas a line with an end bar indicates inhibition. Abbreviations: AP-1, activating protein-1 transcription factor; ETC, electron transport chain; H1R, histaminergic 1 receptor; H_2_O_2_, hydrogen peroxide; H4R, histaminergic 4 receptor; HA, histamine; HDC, histidine decarboxylase; iNOS, induced nitric oxide synthase; MAO-B, monoamine oxidase B; mtDNA, mitochondrial deoxyribonucleic acid; N-MIAA, N-methyl indole acetic acid; NF-kappaB, nuclear factor kappa-light-chain-enhancer of activated B cells; NO, nitric oxide; NOX, NADPH oxidase; OXPHOS, oxidative phosphorylation; ROS, reactive oxygen species; TCA cycle, tricarboxylic acid cycle; VMAT2, vesicular monoamine transporter 2.

### 5.5 Histaminergic system and mitochondrial dysfunction in AD

The histaminergic system originates from the tuberomammillary nucleus (TMN) at the posterior hypothalamus and projects to diverse brain regions such as the neocortex, thalamus, basal ganglia, amygdala, and hippocampus [247]. Histamine levels in the brain are associated with cognition, attention, learning, memory, sensory, and motor functioning [248, 249]. Although histaminergic neurones in the TMN are known to be affected in the early stages of AD [250-252], the changes in HA levels have varied across studies. Some studies reported a reduction in HA levels in the hypothalamus, hippocampus, and temporal cortex of post-mortem AD brains [252-254], whereas one study reported elevated levels in the cortical and subcortical structures, except the globus pallidus and corpus callosum [247]. This discrepancy might be due to elevated HA production from microglia in the central nervous system [255]. Unlike cholinergic and other monoaminergic systems, to our knowledge, the relationship between the mitochondrial-mediated apoptotic pathway and neuronal death in the TMN is still unclear.

#### 5.5.1 HA activates microglia-mediated mitochondrial dysfunction

Histamine is an important inflammatory modulator that causes oxidative stress and mitochondrial dysfunction at pre- and postsynaptic terminals. It activates HA 1 and 4 receptors on microglial membranes to activate the NADPH oxidase signalling pathway and ROS production [256], leading to possible mtDNA damage. Moreover, HA also induces NO synthesis in microglia by upregulating iNOS via extracellular signalling to activate transcription factors like NF-KappaB and activator protein 1 [257]. The iNOS-induced NO can stimulate glutamate excitotoxicity and modify proteins by nitrosylation and nitrosation [258, 259]. Overall, HA induces ROS and NO in microglia that promotes oxidative stress and mitochondrial damage.

## 6. Neuroinflammation, mitochondrial dysfunction, and neurotransmission failure

Another important mechanism is that mitochondrial dysfunction mediates neuroinflammation, which possibly leads to synaptic deficits. An important source of neuroinflammation in neurodegenerative diseases is via mtDNA damage. Evidence indicates that mtDNA damage, which can be non-specific and specific, activates the canonical inflammatory pathway by stimulating both microglia and astrocytes [5, 260]. This eventually leads to increased expression of proinflammatory cytokines such as tumour necrosis factor-α, interleukin (IL)-1β, IL-6, and IL-10, as well as nuclear factor-κB and toll-like receptors [261]. Stimulated microglia increase ROS and reactive nitrogen species levels, which subsequently activates glial inflammatory mechanisms [262]. The oxidative stress from mitochondrial dysfunction is potentiated by microglial-mediated oxidative stress, which pathologically induces oxidative damage in mtDNA, which again activates the above process [262]. Therefore, neuroinflammation likely occurs and is amplified due to mtDNA damage and oxidative stress, which are predominant in mitochondria in AD.

**Table 2:** Common mitochondrial dysfunctions associated with neurotransmission in Alzheimer’s disease.

Mitochondrial dysfunctions	Acetylcholine	Serotonin	Dopamine	Norepinephrine	Histamine
TCA cycle disturbance	++	+	N.A.	N.A.	+?
ETC impairment	+?	+	+	+	+?
Oxidative stress	+?	+?	+?	+	+
mtDNA damage	N.A.	N.A.	+	+	+?
Ca^2+^ dysregulation	+	N.A.	N.A.	+	+
Morphological change	N.A.	N.A.	+	+	N.A.
Transportation dysfunction	N.A.	+	+?	N.A.	N.A.
Membrane permeabilisation	++	+	+?	++	+?

The ‘+’ indicates that the mitochondrial dysfunction is relevant and ‘++’ indicates that the mitochondrial dysfunction is particularly relevant, with neurotransmission in Alzheimer’s disease. A ‘?’ indicates that the complete mechanism has not been clearly identified. Abbreviations: Ca^2+^, calcium ions; ETC, electron transport chain; N.A., not avaliable; mtDNA, mitochondrial deoxyribonucleic acid; TCA, tricarboxylic acid.

Upon neuroinflammation, the axonal transport of mitochondria, especially retrograde trafficking, is disrupted [263]. This causes local energy depletion, which triggers impaired synaptic vesicle release and neurotransmission failure [44, 264], leading to cognitive deficits in AD. Overall, there is increasing evidence to support neurotransmitter deficits In AD due to mitochondrial dysregulation (oxidative stress and mtDNA damage) and induced neuroinflammation. However, so far there is no evidence of a system-specific association, which needs to be investigated.

## 7. Conclusion and future perspectives

Mitochondrial impairments in AD appear to occur upstream and downstream simultaneously. In fact, it appears that mitochondrial dysfunctions are connected to defective synaptic transmission and leads to changes in cholinergic and monoaminergic systems. Neuro-transmission dysfunctions, such as 5-HT and DA deficit, also affect mitochondrial trafficking. All these pathological events act as a self-fuelling vicious cycle which ultimately leads to the disease progression in AD patients, culminating in clinical symptoms of memory deficit, depression, anxiety, and agitation. Table 2 summarizes the relationships reported in the literature and possible mechanisms that have not yet been proven or require further study.

Our current understanding not only sheds light on the pathological mechanisms, but also gives us clues for potential treatments that emphasise mitochondrial protection. In addition to biologics, drug and gene therapies, the major therapeutic approaches appear to be caloric restriction and exercise. Caloric restriction has been shown to reduce ROS and improve ATP/ROS ratio in mitochondria [265]. Exercise induces mitohormesis [266] and improves cognitive functions by promoting neurogenesis and synaptogenesis [267]. Available biologics such as engineered human mitochondrial transcription factor A has been shown to inhibit Aβ aggregation [268] and protect mtDNA function to improve cognitive function in animal models [269]. Drug therapies including mitochondrial-targeting bioenergetics (e.g., mitovitE, coenzyme Q10) can potentially rescue mitochondrial functions and improve AD conditions [270]. Recently, mtDNA functions in AD have been restored through gene therapy techniques called transcription activator-like effector nucleases and clustered regularly interspaced short palindromic repeats / associated protein 9 (CRISPR/Cas9) technology [110]. Although all of these approaches can improve mitochondrial dysfunction and AD pathology, none of them can completely rescue the condition.

A more accurate characterisation of the mechanisms underlying the complicated cross-talk between mitochondrial dysfunction and failure of cholinergic and monoaminergic systems could lead to a better understanding of how mitochondrial dysfunction affects the symptomology of AD, which could advance our knowledge of the molecular mechanisms and facilitate neuroprotective strategies aimed at interrupting the mitochondrial cascade to successfully treat patients with AD.
